# Techniques to tell the real story: narrative inquiry in health services research

**DOI:** 10.1186/1472-6963-14-S2-P22

**Published:** 2014-07-07

**Authors:** Nicole Ison, Anne Cusick, Rosalind A Bye

**Affiliations:** 1 National Disability Coordination Program, South Western Sydney, Department of Education, Australian Government, Penrith, NSW, Australia; 2 School of Health and Society, University of Wollongong, Wollongong, NSW, Australia; 3 Occupational Therapy, University of Western Sydney, Penrith, NSW, Australia

## Background

Fifteen years ago in Health Services Research (1999) qualitative research methods were argued to be useful and valid. Since that time qualitative research methods have gained increasing legitimacy however qualitative research papers remain underrepresented in high impact health journals [1]. The rigour of qualitative methods and their relevance in policy evaluation and development is, however, a continuing debate. On the one hand, qualitative inquiry methods bring the complexity of health service policy impacts to the fore; they provide policy makers with perspectives from the people services aim to support. On the other hand, the variability of qualitative approaches can lead to questions around validity and utility of findings. Narrative inquiry is one qualitative approach which, like others, has no prescribed method. Yet it is a method gaining increasing popularity in social science, clinical and health services research. This paper makes the methodological case for narrative inquiry in health services research and recommends techniques.

## Narrative inquiry in health service research

Narrative inquiry is based on the premise that by listening to the stories of others we can make sense of their experience and understand how they construct meaning within a broader social context. Data is usually collected through interview. Data analysis includes narrative analysis and/or paradigmatic analysis of narratives [2]. Narrative analysis produces an individual story for each participant while paradigmatic analysis of narratives identifies a typology of story types.

## Cerebral palsy health service research: a case study

We used narrative inquiry to explore health service experiences of 18 young adults with cerebral palsy. It is important to select a scholarly framework to guide but not constrain the inquiry. We selected Polkinghorne [2]. We collected data through multiple in-depth, unstructured interviews using face-to-face, assistive technology for augmented communication, phone and email. Data was transcribed. Narrative and paradigmatic analysis was used. The narrative approach revealed 18 individual health service stories and four over-arching narratives that captured the depth of unique individual experience at the same time as a breadth of common themes.

The techniques and processes used in this study can be replicated and transparently reported thus demonstrating the rigour required for narrative inquiry in health services research.
